# Heterogeneous nuclear ribonucleoprotein A1 regulates rhythmic synthesis of mouse Nfil3 protein via IRES-mediated translation

**DOI:** 10.1038/srep42882

**Published:** 2017-02-21

**Authors:** Hyo-Jin Kim, Hwa-Rim Lee, Ji-Young Seo, Hye Guk Ryu, Kyung-Ha Lee, Do-Yeon Kim, Kyong-Tai Kim

**Affiliations:** 1 Department of Life Sciences, Pohang University of Science and Technology (POSTECH), Pohang, Gyeongbuk, Korea; 2 School of Interdisciplinary Bioscience and Bioengineering, Pohang University of Science and Technology (POSTECH), Pohang, Gyeongbuk, Korea; 3 Division of Integrative Biosciences and Biotechnology, Pohang University of Science and Technology (POSTECH), Pohang, Gyeongbuk, Korea; 4 Division of Bio-Technology and Convergence, Daegu Haany University, Gyeongsan, Gyeongsangbuk-do, Korea; 5 Department of Pharmacology, School of Dentistry, Kyungpook National University, Daegu, Korea

## Abstract

Nuclear factor, interleukin 3, regulated (Nfil3, also known as E4 Promoter-Binding Protein 4 (E4BP4)) protein is a transcription factor that binds to DNA and generally represses target gene expression. In the circadian clock system, Nfil3 binds to a D-box element residing in the promoter of clock genes and contributes to their robust oscillation. Here, we show that the 5′-untranslated region (5′-UTR) of Nfil3 mRNA contains an internal ribosome entry site (IRES) and that IRES-mediated translation occurs in a phase-dependent manner. We demonstrate that heterogeneous nuclear ribonucleoprotein A1 (hnRNP A1) binds to a specific region of Nfil3 mRNA and regulates IRES-mediated translation. Knockdown of hnRNP A1 almost completely abolishes protein oscillation without affecting mRNA oscillation. Moreover, we observe that intracellular calcium levels, which are closely related to bone formation, depend on Nfil3 levels in osteoblast cell lines. We suggest that the 5′-UTR mediated cap-independent translation of Nfil3 mRNA contributes to the rhythmic expression of Nfil3 by interacting with the RNA binding protein hnRNP A1. These data provide new evidence that the posttranscriptional regulation of clock gene expression is important during bone metabolism.

The circadian (24 hour) clock system is present in organisms ranging from single-cell organisms such as cyanobacteria to multi-cell organisms such as mammals^1^,^2^,^3^. In mammals, the suprachiasmatic nucleus (SCN) of the anterior hypothalamus is the circadian pacemaker that synchronizes rhythm in the brain and peripheral tissues, including the musculoskeletal system^4^,^5^. This synchronization leads to circadian rhythmicity of clock genes as well as biological physiology and behavior^6^. The mammalian circadian rhythm is composed of networks of transcriptional-translational feedback loops of core clock genes^7^,^8^. The basic helix-loop-helix transcription factors Clock and Bmal1 form a heterodimer and actively regulate the transcription of core clock genes such as Periods (Per) and Cryptochromes (Cry) by binding to their E-box elements (CAGGTG). The translated Per and Cry form a heterodimer that translocates to the nucleus. This complex binds to the Clock-Bmal1 heterodimer and inhibits its transcriptional activity^9^. This network of negative-feedback loop is necessary for the tight regulation of clock gene expression.

Nuclear factor, interleukin 3, regulated (Nfil3, also known as E4 Promoter-Binding Protein 4 (E4BP4)), was first identified as an interleukin-3 (IL-3) induced nuclear factor in pro-B lymphocytes^10^,^11^. Nfil3 is a basic leucine zipper transcription factor^12^ that binds to a D-box element ([G/A]T[G/T]A[C/T]GTAA[C/T])^13^. Nfil3 is important in the immune system, for example during NK cell development and IgE class switching^14^,^15^. In DRG neurons, Nfil3 plays the role of transcriptional regulator of CREB and C/EBP, which are proteins that contribute to neuroregeneration and neuronal outgrowth^16^,^17^. In *Drosophila*, the Nfil3 homologue *vrille* constitutes a negative feedback loop of clock gene expression^18^,^19^. In mammals, Nfil3 binds to D-box elements residing in the promoters of clock genes such as Period. Nfil3 negatively regulates the transcription of these genes by competing with proline-alanine rich (PAR) proteins such as DBP, HLF and TEF, in an anti-phasic oscillatory manner^20^,^21^. Additionally, Nfil3 targets clock-controlled genes (CCGs)^13^,^22^,^23^,^24^ and represses their transcription. Although important roles for Nfil3 have been demonstrated in several physiological conditions, the regulatory mechanism underlying Nfil3 expression remains unclear.

To date, the maintenance and robustness of clock genes have been studied at the level of transcription, translation and posttranslational regulation^8^. There has been growing evidence suggesting that posttranscriptional regulation may contribute to the fine-tuning of gene expression, but this regulation is not as that well understood compared to some other mechanisms^25^,^26^. Specifically, the regulation of phase-dependent translational initiation is known to contribute to the robust rhythmic biosynthesis of clock gene proteins. Because Nfil3 protein regulates D-box-containing clock genes, the investigation of the translation mechanism of Nfil3 mRNA could reveal the importance of posttranscriptional regulation of clock genes. Here, we suggest that mouse Nfil3 mRNA is translated in an internal ribosome entry site (IRES) -dependent manner in MC3T3-E1 mouse osteoblast cells. IRES was first discovered in the viral genome^27^,^28^. During IRES-mediated regulation, ribosomes are recruited directly to the 5′-UTR to process translation in a cap-independent manner^29^. Moreover, previous studies have suggested that cellular IRES-mediated translation occurs under specific stress conditions^30^,^31^,^32^ and is required for robust oscillation of clock proteins^33^,^34^,^35^, which consolidates our suggested mechanisms.

In the present study, we showed that the α_1_-adrenergic receptor agonist phenylephrine (PHE) synchronizes and drives mouse Nfil3 oscillation in MC3T3-E1 mouse osteoblast cells. We found that Nfil3 mRNA contains an IRES element in the 5′-UTR, and that IRES-mediated translation is critical for maintaining Nfil3 protein oscillation. We had also identified an RNA binding protein hnRNP A1 that specifically binds to the IRES element of Nfil3 5′-UTR, and show that hnRNP A1 has a crucial role in the IRES-mediated translation of Nfil3 mRNA and oscillation of Nfil3 protein. Finally, we observed that Nfil3 translation regulates intracellular calcium levels in osteoblast cells.

## Results

### Nfil3 protein and mRNA oscillation following phenylephrine treatment

**Figure 1:**
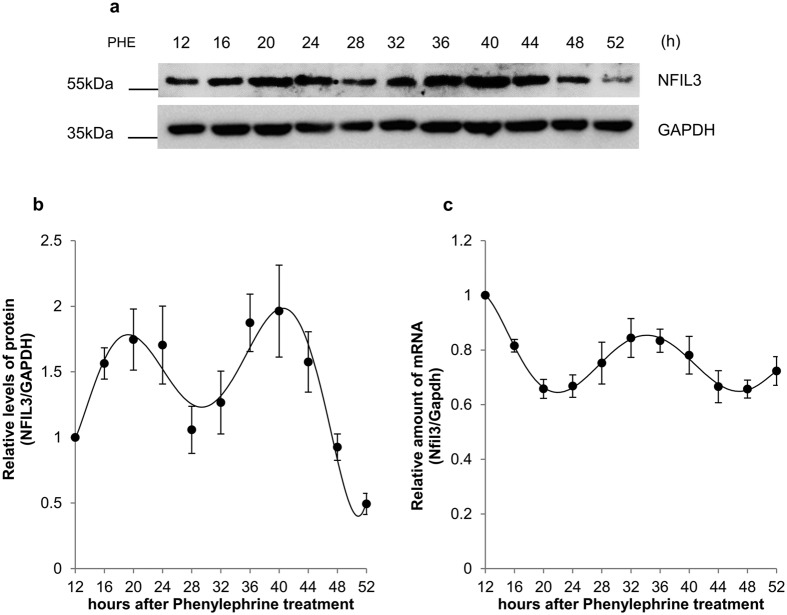
Nfil3 has rhythmic expression in MC3T3-E1 osteoblasts. (**a**) Oscillation of Nfil3 protein shown by immunoblotting (IB). MC3T3-E1 cells were treated with 10 μM phenylephrine (PHE), then harvested at the indicated time points. Whole cell lysates were subjected to immunoblotting. This result is representative of four independent experiments. Full-length blots are presented in Figure S1 and band of interest is indicated by a red box. (**b**) Expression profile of Nfil3 protein following phenylephrine treatment. Relative levels of Nfil3 protein were normalized to Gapdh protein, then calculated and plotted. The initial level of Nfil3 protein was arbitrarily set to 1.0. Error bars represent mean ± SEM (*n* = 4). (**c**) Expression profile of Nfil3 mRNA following phenylephrine treatment. MC3T3-E1 cells for mRNA oscillation measurement were prepared in panel (**a**) were subjected to quantitative real-time PCR. Analysis of mRNAs was performed using specific primers. Relative levels of Nfil3 mRNA were normalized to Gapdh mRNA. The initial Nfil3 mRNA level was arbitrarily set to 1.0. Error bars represent mean ± SEM (*n* = 4).

α_1_-adrenergic receptor (α_1_-AR) is a member of the G protein-coupled receptor (GPCR) superfamily of membrane proteins, which consists of 9 highly homologous subtypes. α_1_-AR plays key roles in diverse mechanisms, such as neurological functions during locomotive activity, regulation of muscle contraction and growth response^36^. A recent study demonstrated that signaling via α_1_-AR is also responsible for the rhythmic mRNA expression of clock genes, including Nfil3^37^. Therefore, first, we investigated the time-dependent oscillation of Nfil3 protein in response to activation of α_1_-AR signaling. We treated MC3T3-E1 osteoblast cells with phenylephrine, a selective α_1_-AR agonist, and observed robust oscillation of Nfil3 protein (Fig. 1a and b, Figure S1) and mRNA (Fig. 1c) expression levels. The oscillation of mRNA levels is in agreement with results from a previous study^37^. In addition to Nfil3, we examined the mRNA of core clock genes and confirmed their rhythmic and oscillatory expression after phenylephrine treatment (Figure S2).

### Existence of an IRES region in the Nfil3 5′-UTR

mTOR is a serine/threonine protein kinase that is thought to regulate several cellular signaling networks, including protein synthesis^38^. Rapamycin inhibits the mTOR pathway by inducing hypophosphorylation of eukaryotic initiation factor 4E binding protein (4E-BP). This modification increases the binding between eukaryotic initiation factor 4E (eIF4E) and 4E-BP, and impairs ribosome recruitment to the cap-structure of mRNA, which then decreases translation^38^. The exposure of MC3T3-E1 cells to rapamycin also interferes with the phosphorylation of S6 ribosomal protein, which is a component of the 40 S ribosomal subunit that is phosphorylated by p70-S6 kinase 1 (S6K1)^39^. We treated the mouse osteoblast cells with rapamycin to inhibit canonical cap-dependent translation. The phosphorylation of S6 ribosomal protein decreased at 4 hours after rapamycin treatment, while the level of Nfil3 did not change. On the other hand, we observed a dramatic degradation of Nfil3 after the treatment with the translation elongation inhibitor cycloheximide (CHX), which binds to ribosomes and inhibits translocation (Figure S3). These results suggest that Nfil3 could be generated by both cap-independent and -dependent translations.

**Figure 2:**
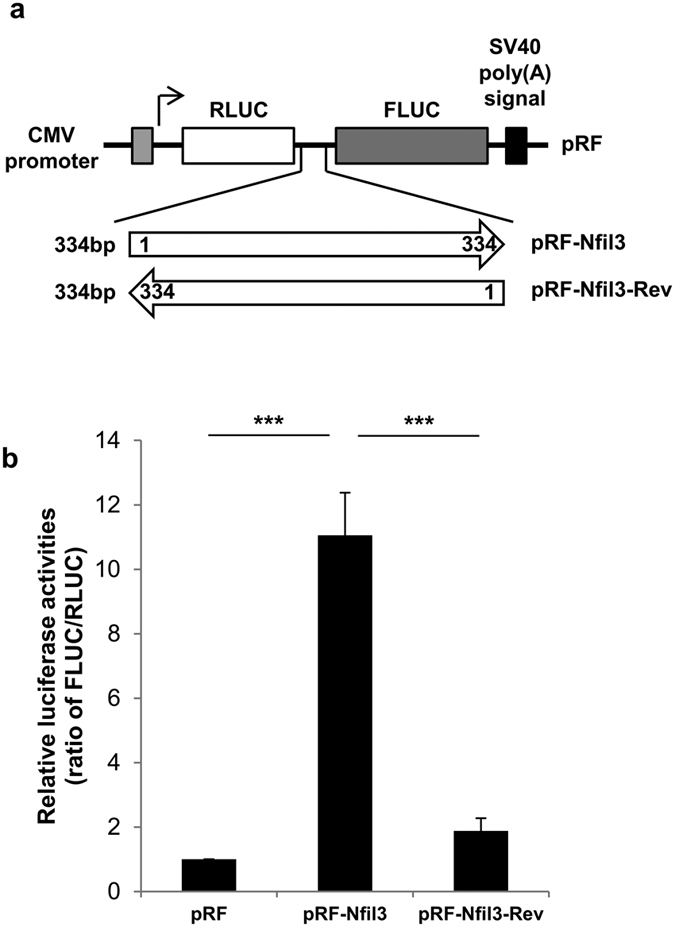
IRES-mediated translation of Nfil3. (**a**) Schematic diagram of the bicistronic reporter vector containing the full-length Nfil3 5′-UTR. In the pRF vector, the Nfil3 5′-UTR sequence was inserted between *Renilla* luciferase and firefly luciferase. Inverse sequences of Nfil3 5′-UTR are also shown. SV40, simian virus 40. (**b**) MC3T3-E1 cells were transfected with the bicistronic reporter constructs and incubated for 24 hours. Cells were harvested at the indicated times, and cell lysates were subjected to a luciferase assay. Graph shows the ratio of relative luciferase activities, FLUC/RLUC. The ratio of empty vector (pRF) was arbitrarily set to 1.0. Error bars represent mean ± SEM (*n* = 3), *** *P* < 0.001.

To identify IRES-mediated translation, we introduced the bicistronic luciferase reporter vector system to the MC3T3-E1 cells. An IRES is commonly located in the 5′-UTR; therefore, we inserted mouse Nfil3 5′-UTR sequences between the *Renilla* luciferase (Rluc) and firefly luciferase (Fluc) coding sequences (Fig. 2a). While Rluc, upstream of Nfil3 5′-UTR, is translated in a cap-dependent manner, the translation of Fluc depends on the IRES insertion upstream of Fluc. To exclude nonspecific effects due to 5′-UTR length, we also inserted inverted Nfil3 5′-UTR sequences into the reporter vector (Fig. 2a). Interestingly, the Nfil3 5′-UTR enhanced Fluc translation approximately 10-fold compared to the control vector. In contrast, the inverted Nfil3 5′-UTR increased Fluc translation by less than 2-fold (Fig. 2b). These results suggest that the mouse Nfil3 5′-UTR contains potent IRES elements.

To exclude the possibility that cryptic promoter activity in the Nfil3 5′-UTR initiates the transcription of Fluc, we deleted the CMV promoter from the reporter vector (Figure S4a). In promoter deletion constructs, Rluc and Fluc were expressed at a basal level (Figure S4c and d). Next, to prevent ribosome re-initiation of ribosomes that were not released from the stop codon of Rluc and instead became reloaded to the Fluc start codon then translated, we inserted a hairpin loop upstream of Rluc (Figure S4b). We observed that insertion of hairpin reduced Rluc (first cistron) expression by 50% compared to pRF-Nfil3, while Fluc (second cistron) expression was not affected (Figure S4c and d). These data show that there is no cryptic promoter activity in the Nfil3 5′-UTR and that the translation of Fluc was affected only by the IRES. These results indicate that the mouse Nfil3 5′-UTR contains an IRES, which may initiate translation in a cap-independent manner.

In case of cellular IRES, some IRES sequences were conserved among species^40^. Thus, we proceed to test whether human Nfil3 5′-UTR also shows an IRES activity or not. Currently three variants human 5′-UTR have been reported, and interestingly, one of variants has an IRES activity and the mouse Nfil3 5′-UTR shows approximately 60% of sequence similarity compared to this variant (Figure S5a and b). This results indicate that conserved IRES elements in Nfil3 5′-UTR may be important during the translation of human and mouse Nfil3 mRNA.

### Identification of a specific IRES region in the Nfil3 5′-UTR

**Figure 3:**
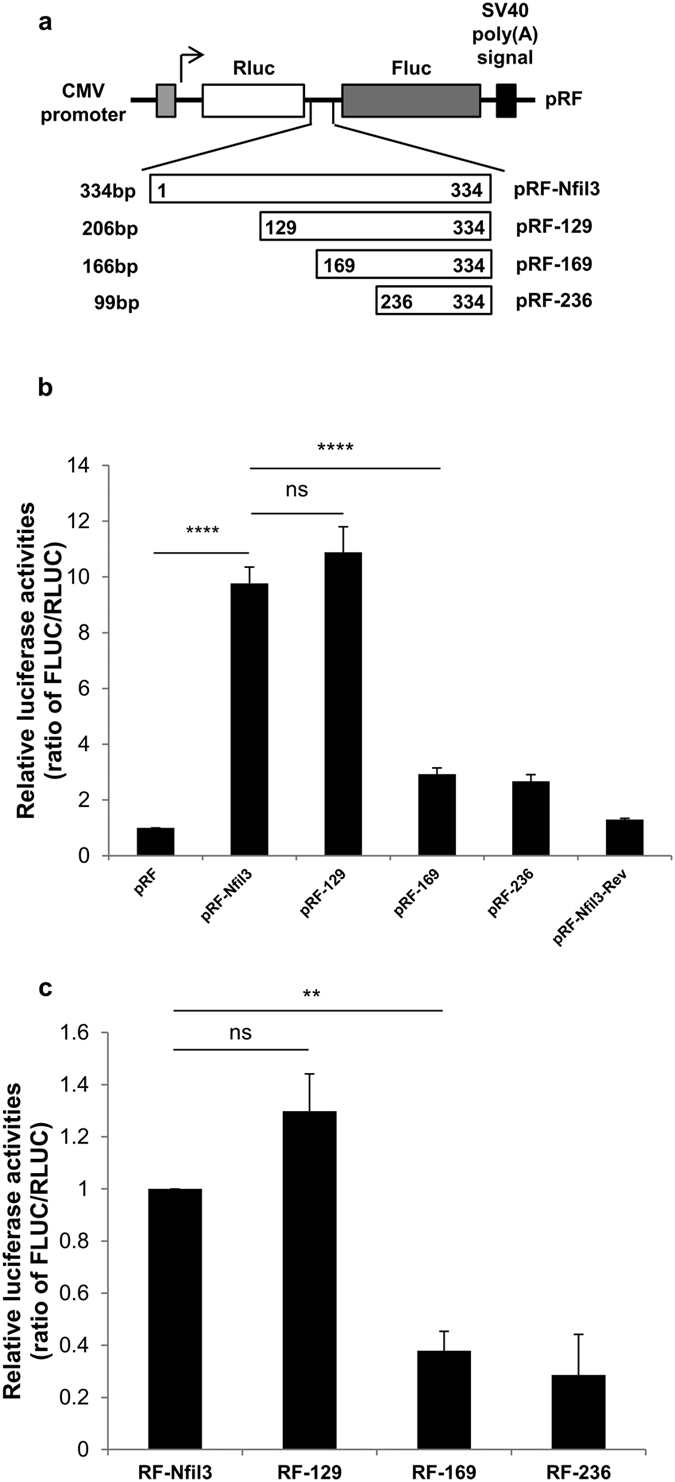
Determination of a *cis*-element in Nfil3 IRES. (**a**) Schematic diagram of Nfil3 5′-UTR deletion constructs. (**b**) MC3T3-E1 cells were transfected with the control and deletion constructs and incubated for 24 hours. Cells were harvested at the indicated times, and cell lysates were subjected to a luciferase assay. The ratio of empty vector (pRF) was set to 1.0. Error bars represent mean ± SEM (*n* = 3), ****P < 0.0001, ns *P* ≥ 0.05. (**c**) *In vitro* transcribed reporter mRNAs containing the Nfil3 5′-UTR were transfected into MC3T3-E1 cells and incubated for 12 hours. Cells were harvested at the indicated times, and cell lysates were subjected to a luciferase assay. The ratio of full-length of Nfil3 (RF-Nfil3) set to 1.0. Error bars represent mean ± SEM (*n* = 4), **P < 0.01, ns *P* ≥ 0.05.

The IRES element recruits ribosomes to initiate translation. It is mediated by the secondary structure of the IRES sequence^41^ and RNA binding proteins that bind specifically to the IRES element. To identify the IRES *cis*-acting element in the Nfil3 5′-UTR that is responsible for ribosome recruitment, we inserted the deleted 5′-UTR fragments between the Rluc and Fluc coding sequences (Fig. 3a). We observed that the remained IRES activity as full-length of Nfil3 5′-UTR when 128 nucleotides were deleted from the 5′ end (pRF-129). Conversely, we observed a dramatic reduction in IRES activity when 168 nucleotides (pRF-169) or 235 nucleotides (pRF-236) were deleted, which showed approximately 30% IRES activity compared to the full-length 5′-UTR (Fig. 3b). Next, we transfected MC3T3-E1 cells with transcribed reporter mRNA. This excludes ribosome re-initiation and cryptic promoter activity, and therefore, provides conclusive evidence for the existence of an IRES in the Nfil3 5′-UTR. The mRNA transfection assay also caused a dramatic downregulation in Fluc levels with the 168- and 235-nucleotide deletion constructs compared to the full-length and 128-nucleotide deletion constructs, as well as DNA transfection (Fig. 3c). These results suggest that the region between nucleotides 129 and 169 in the Nfil3 5′-UTR is critical for IRES-mediated translation.

### hnRNP A1 regulates IRES-mediated translation via a direct interaction with the Nfil3 5′-UTR

**Figure 4:**
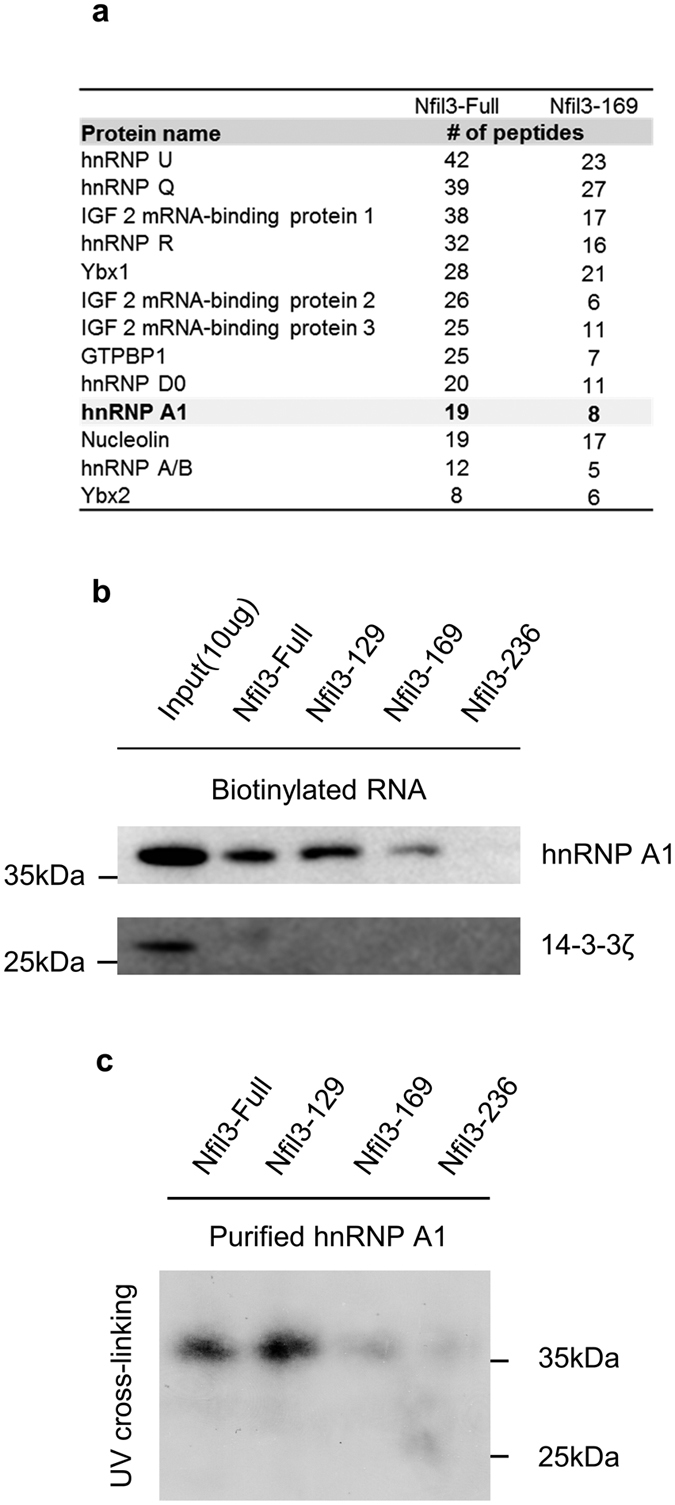
hnRNP A1 directly binds to the Nfil3 5′-UTR. (**a**) RNA interacting proteins that isolated by biotin-conjugated RNA pull-down using streptavidin beads were analyzed by LTQ-orbitrap. The identified proteins are listed, along with the number of peptides found after mass-spectrometry analysis. (**b**) Confirmation of the interaction between the *cis*-element of the Nfil3 IRES and hnRNP A1 using biotin-conjugated RNA pull-down followed by immunoblotting. Cytoplasmic cell lysates incubate with biotin-conjugated full-length and deletion constructs of Nfil3 5′-UTR and streptavidin beads. Streptavidin-affinity purified samples were separated by SDS-PAGE and subjected to immunoblotting with specific antibidies. 14-3-3 ζ was used as a negative control for the RNA pull-down analysis. Full-length blots are presented in Figure S6 and band of interest is indicated by a red box. (**c**) To prove direct interaction of hnRNP A1 and Nfil3 5′-UTR, [^32^P]UTP-labeled Nfil3 5′-UTR RNAs were prepared by *in vitro* transcription and subjected to UV cross-linking assay with a non-tagged purified hnRNP A1 and autoradiographic intensities were checked. A 38 kDa protein whose size is the same as hnRNP A1. Full-length blot is presented in Figure S7 and band of interest is indicated by a red box.

IRES *trans*-acting factors (ITAFs), which bind to the IRES *cis*-acting element in a recognition- and sequence-specific manner, are required for IRES-mediated translation. ITAFs directly recruit ribosomes to the IRES and can modulate cap-independent translation^42^. To identify ITAF candidates, we performed an *in vitro* biotinylation assay using *in vitro* transcribed, biotin-conjugated full-length constructs of Nfil3 5′-UTR that were pulled-down from MC3T3-E1 cytoplasmic cell lysates. Using mass-spectrometry we identified several putative RNA binding proteins that interact with the full-length of Nfil3 5′-UTR and Nfil3-169 construct, which shows a dramatically reduced IRES activity, analyzed together (Fig. 4a). After analyzing these ITAF candidates we did not observe any changes in IRES activity in the bicistronic reporter system using siRNA. It is possible that the candidates are required for formation of the RNA binding protein complex and are involved in other posttranscriptional regulation processes. However we identified heterogeneous nuclear ribonucleoprotein A1 (hnRNP A1) as a strong candidate protein for the ITAF involved in this system.

To prove that hnRNP A1 is an ITAF for IRES-mediated translation of Nfil3, we confirmed the interaction between hnRNP A1 and the Nfil3 5′-UTR using biotinylation assay and immunoblot (Fig. 4b, Figure S6). We found a dramatic decrease in the interaction between hnRNP A1 and the deleted Nfil3-169 and Nfil3-236 constructs. This is in agreement with our previous finding that the region between 129 and 169 in the Nfil3 5′-UTR acts as a *cis-*acting element. It is possible that the result of the *in vitro* biotinylation assay is due to an indirect RNA-protein interaction; thus requiring us to determine whether hnRNP A1 binds directly to the Nfil3 5′-UTR or not. To test this, we performed a UV cross-linking assay using *in vitro* purified hnRNP A1 protein (Fig. 4c, Figure S7). The non-tagged purified hnRNP A1 bound to the full-length and Nfil3-129 constructs, but not to the Nfil3-169 or Nfil3-236 constructs. These data identify hnRNP A1 as a strong candidate for the ITAF that interacts with the *cis*-acting element of the Nfil3 5′-UTR.

### hnRNP A1 is a crucial ITAF in the IRES-mediated translation of Nfil3

**Figure 5:**
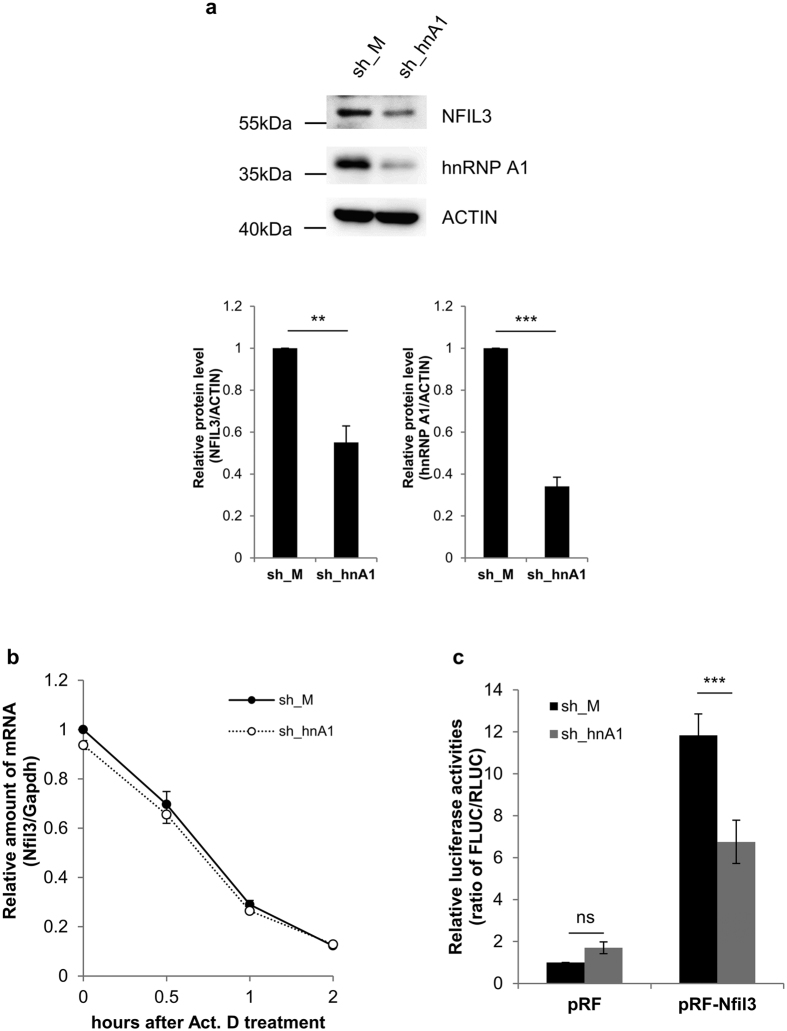
hnRNP A1 is ITAF of Nfil3 IRES-mediated translation. (**a**) A shRNA generating vector for hnRNP A1 (sh_hnA1) and empty vector (sh_M) were transiently transfected into MC3T3-E1 cells. To confirm hnRNP A1 knockdown, cells were harvested at 48 hours after transfection and subjected to immunoblotting. Relative levels of Nfil3 and hnRNP A1 protein were normalized to Actin protein. Protein levels in sh_M tranfected cells were set to 1.0. Error bars represent mean ± SEM (*n* = 3), ***P < 0.001, **P < 0.01. Full-length blots are presented in Figure S8 and band of interest is indicated by a red box. (**b**) MC3T3-E1 cells that transfected as in Fig. 5a, treated with actinomycin D and harvested at indicated time points than subjected to quantitative real-time PCR to measure Nfil3 mRNA stabilities. Relative sh_M (closed circles/solid line) and sh_hnA1 (open circles/dotted line) transfected Nfil3 mRNA were normalized to Gapdh mRNA. The initial mRNA level in sh_M transfected cells set to 1.0. To statistically analyze the mRNA stability between sh_M and sh_hnA1, a two-way ANOVA was performed. Error bars represent mean ± SEM (*n* = 3). ns *P* ≥ 0.05. (**c**) MC3T3-E1 cells were transfected with sh_M and sh_hnA1 expression vectors before the transfection with a bicistronic reporter vector. Samples were harversted 24 hours after transfection of the reporter vector, and subjected to immunoblotting and a luciferase assay. The ratio of FLUC/RLUC of cells transfected with sh_M and pRF empty vector was set to 1.0. Error bars represent mean ± SEM (*n* = 5), ***P < 0.001, ns *P* ≥ 0.05.

hnRNP A1 is known to play a role in transcription^43^ and posttranscriptional regulation, such as mRNA turnover^44^, mRNA splicing^45^, translation and miRNA processing^46^. Moreover, hnRNP A1 is also reported to act as an ITAF during cap-independent translation^47^,^48^,^49^. As described earlier, binding of hnRNP A1 to the Nfil3 5′-UTR was decreased when the Nfil3 *cis*-acting region, containing the IRES element, was deleted. To verify the role of hnRNP A1 in the cap-independent translation of Nfil3, we designed small-hairpin RNA (shRNA) to transiently knockdown hnRNP A1. This shRNA against hnRNP A1 (sh_hnA1) significantly reduced endogenous hnRNP A1 protein level and appeared to reduce endogenous Nfil3 protein level (Fig. 5a, Figure S8). To exclude the possibility that hnRNP A1 was affecting Nfil3 mRNA stability, we treated cells with actinomycin D (Act.D) which transcription inhibitor and measured endogenous Nfil3 mRNA level in the presence of sh_hnA1. After Act.D treatment, Nfil3 mRNA level was dramatically decreased but decay kinetics was unaffected by hnRNP A1 knockdown (Fig. 5b). Moreover, the IRES activity of Nfil3 was reduced by about half following knockdown of hnRNP A1 (Fig. 5c), and was increased following overexpression of hnRNP A1 (Figure S9). These results suggest that hnRNP A1 acts as a crucial ITAF during the IRES-mediated translation of Nfil3 mRNA.

### hnRNP A1 is critical for maintaining Nfil3 protein oscillation without altering mRNA oscillation

**Figure 6:**
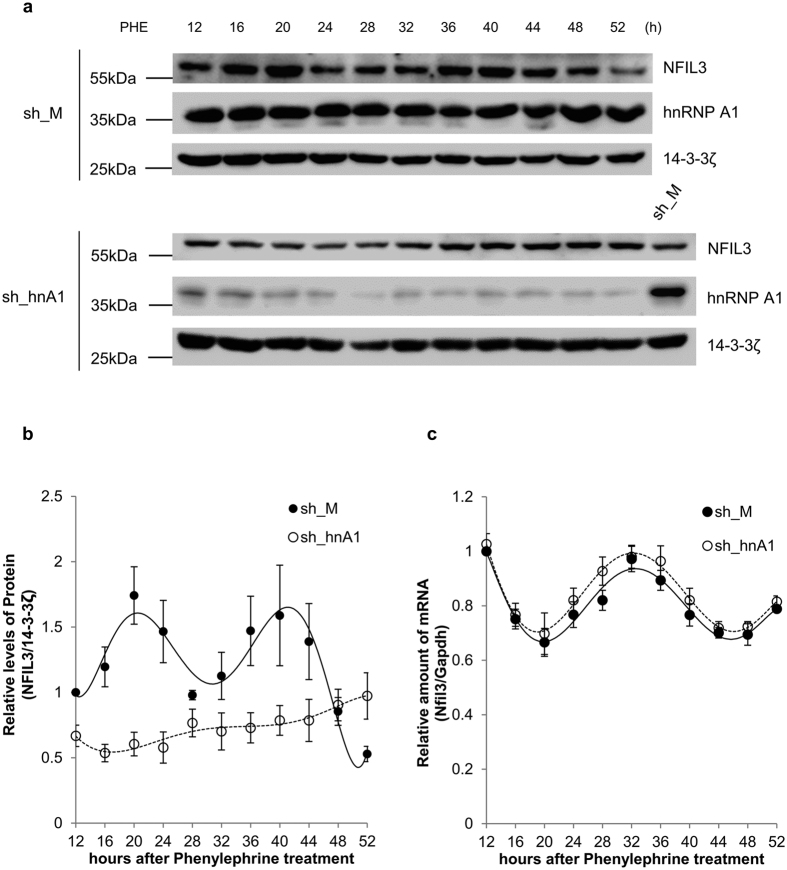
hnRNP A1 regulates Nfil3 protein but not RNA oscillation. (**a**) MC3T3-E1 cells were transfected with sh_M or sh_hnA1 expression vectors, incubated for 48 hours, treated with phenylephrine and harvested at the indicated time points. Samples were subjected to immunoblotting with the indicated antibodies. This result is representative of four independent experiments. Full-length blots are presented in Figure S10 and band of interest is indicated by a red box. (**b**) Relative sh_M (closed circles/solid line) and sh_hnA1 (open circles/dotted line) transfected Nfil3 protein were normalized to 14-3-3ζ protein, calculated and plotted. The ratio of sh_M transfected Nfil3/Gapdh protein at 12 hours after phenylephrine treatment was set to 1.0. Error bars represent mean ± SEM (*n* = 4). (**c**) MC3T3-E1 cells prepared in panel (**a**) were used for RNA quantification. Reverse-transcription and quantitative real-time PCR were performed using specific primers. Relative sh_M (closed circles/solid line) and sh_hnA1 (open circles/dotted line) transfected Nfil3 mRNA were normalized to Gapdh mRNA. The sh_M transfected Nfil3 mRNA level at 12 hours after phenylephrine treatment was set to 1.0. To statistically analyze the oscillation between sh_M and sh_hnA1, a two-way ANOVA was performed. Error bars represent mean ± SEM (*n* = 4). ns *P* ≥ 0.05.

As shown in Fig. 1, Nfil3 has its own rhythmic oscillation. To test the effect of hnRNP A1 on the oscillation of Nfil3 mRNA and protein, we performed a knockdown experiment in MC3T3-E1 osteoblast cells using shRNA against hnRNP A1. Interestingly, hnRNP A1 knockdown resulted in disruption of Nfil3 protein oscillation (Fig. 6a and b, Figure S10). To exclude the possibility that Nfil3 mRNA oscillation was also altered by hnRNP A1 knockdown, we measured Nfil3 mRNA oscillation under the same conditions. As described above, hnRNP A1 knockdown did not affect endogenous Nfil3 mRNA stability, thus, the disruption in Nfil3 protein oscillation was not due to changes in mRNA oscillation (Fig. 6c). These data suggest that hnRNP A1 is involved in the rhythmic expression of Nfil3 protein but not mRNA oscillation.

### Rhythmic IRES activity and localization of hnRNP A1 to the cytoplasm contribute to Nfil3 protein oscillation

**Figure 7:**
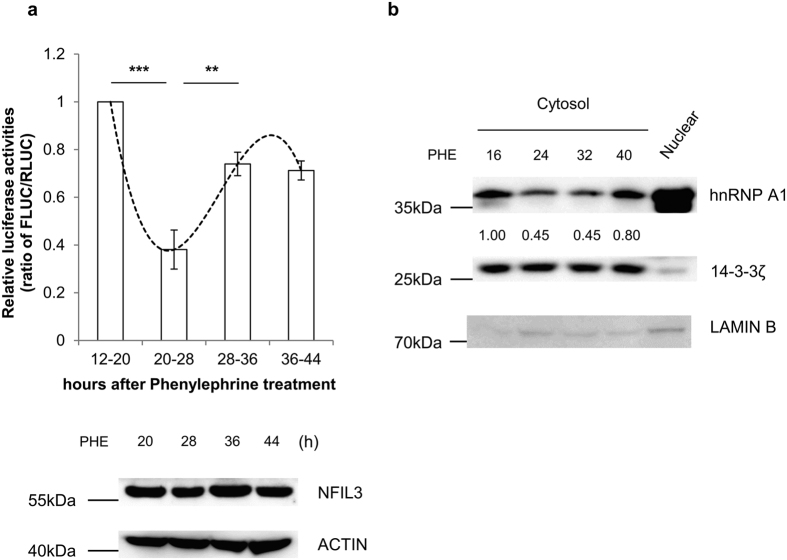
hnRNP A1 contributes to rhythmic IRES-dependent translation of Nfil3. (**a**) MC3T3-E1 cells were treated with 10 μM phenylephrine and transfected 8 hours with *in vitro* transcribed reporter mRNA (RF-Nfil3). Samples were harvested at the indicated times and subjected to a luciferase reporeter assay. Endogenous Nfil3 protein levels at indicated time points shown by immunoblotting. The ratio of FLUC/RLUC of cells transfected 12–20 hours after phenylephrine treatment was set to 1.0. Error bars represent mean ± SEM (*n* = 3), ***P < 0.001, **P < 0.01. Full-length blots are presented in Figure S11 and band of interest is indicated by a red box. (**b**) MC3T3-E1 cells were trated with phenylephrine and harvested at the indicated time points. Cells were lysed separately, and cytosolic and nuclear extracts were subjected to immunoblotting. Changes in cytosolic hnRNP A1 protein level were normalized to 14-3-3ζ protein. The ratio of hnRNP A1/14-3-3ζ protein at 16 hours after phenylephrine treatment was set to 1.0. This result is representative of three independent experiments. Full-length blots are presented in Figure S12 and band of interest is indicated by a red box.

The rhythmic expression of clock genes is thought to be tightly regulated by synthesis and degradation processes^50^. During the rising phase, synthesis is superior to degradation, and this is reversed during the declining phase. We hypothesized that if the oscillation of Nfil3 protein is regulated by IRES-mediated translation, then this IRES activity should also have a rhythmic pattern. To address this hypothesis, we transiently transfected MC3T3-E1 cells with *in vitro* transcribed reporter mRNA in phenylephrine. Interestingly, IRES-mediated translation activity was higher during the rising phases of protein level (12–20 and 28–36 hours) compared to the declining phase (20–28 hours), which is it related to endogenous Nfil3 protein oscillation (Fig. 7a, Figure S11). This result shows that rhythmic IRES-mediated translation of Nfil3 mRNA contributes to Nfil3 protein oscillation.

Next, we sought to determine the relationship between hnRNP A1 and IRES-mediated translation of Nfil3. If rhythmic IRES-mediated translation plays a role, the interaction between the IRES and ITAF should be modulated in a phase-dependent manner^33^,^34^. hnRNP A1 is mainly localized in the nucleus, but has been reported to accumulate in the cytoplasm under osmotic stress, to reduce protein synthesis^49^. Phenylephrine-treated MC3T3-E1 cells were harvested during the rising and declining phases and fractionated. Cytosolic hnRNP A1 protein levels were altered in a phase-dependent manner. During the rising phases (16 and 40 hours), cytosolic hnRNP A1 protein levels were higher than during the declining phases (24 and 32 hours) (Fig. 7b, Figure S12). These results suggest that cytosolic hnRNP A1 protein levels change in a phase-dependent manner, and changes in its localization contribute to the rhythmic IRES-mediated translation of Nfil3 mRNA.

### Nfil3 protein contributes to intracellular calcium concentration

**Figure 8:**
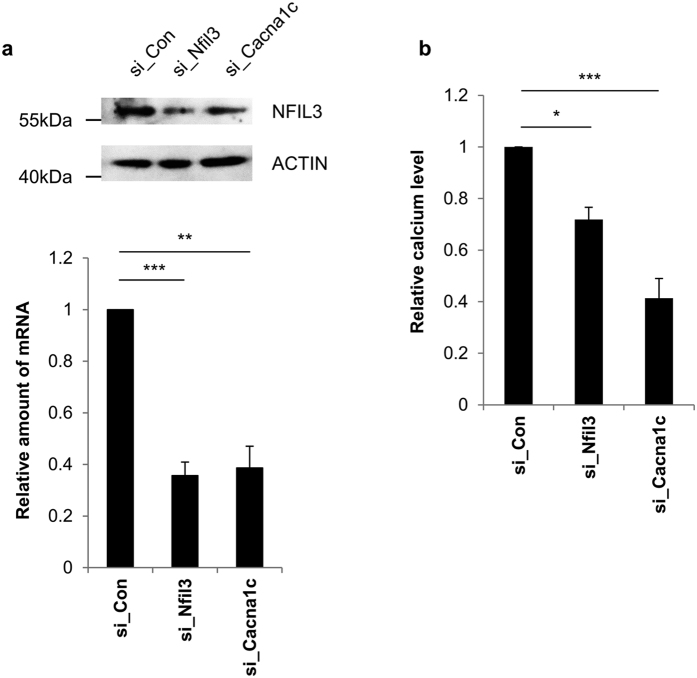
Intracellular calcium level is regulated by Nfil3. (**a**) siRNA against endogenous Nfil3 (si_Nfil3), Cacna1c (si_Cacna1c) or control siRNA (si_Con) were transiently transfected into MC3T3-E1 cells. Transfected cells were harvested at 48 hours after transfection and subjected to immunoblotting or quantitative real-time PCR. Nfil3 and Cacna1c mRNA were quantified specific primers and normalized to Gapdh mRNA. The si_Con transfected Nfil3 and Cacna1c mRNA level were set to 1.0. To statistically analyze the each mRNA level, two-tailed unpaired Student’s *t*-tests was performed. Error bars represent mean ± SEM (*n* = 3), ***P < 0.001, **P < 0.01. si_Cacna1c was used as a positive control for this assay. Full-length blots are presented in Figure S13 and band of interest is indicated by a red box. (**b**) MC3T3-E1 cells prepared in panel (**a**) were subjected to intracellular calcium measurement. The intracellular calcium level in si_Con transfected cells was set to 1.0. Error bars represent mean ± SEM (*n* = 3), ***P < 0.001, *P < 0.05.

Bone is a storage facility for calcium in the body and it is the main calcium source for maintaining extracellular homeostasis. Intracellular calcium levels are related to a number of signaling pathways involved in bone formation and osteoblast differentiation^51^,^52^. Intracellular calcium phosphate in osteoblasts is also linked to bone mineral formation^53^,^54^. We sought to determine whether or not Nfil3 affected osteoblast metabolism via regulation of intracellular calcium levels. First, we assessed the possibility that intracellular calcium level is regulated by Nfil3 protein level using siRNA against endogenous Nfil3. Additionally we used siRNA against the endogenous calcium voltage-gated channel subunit alpha1 C (Cacna1c) as a positive control for this assay. Cacna1c is a primary site for calcium influx in osteoblast^55^. siRNA-mediated knockdown was confirmed by immunoblot and quantitative real-time PCR (Fig. 8a, Figure S13). As expected, intracellular calcium level decreased in Cacna1c knockdown cells and downregulation of Nfil3 protein caused a decrease in intracellular calcium level (Fig. 8b). We also measured intracellular calcium level following hnRNP A1 knockdown and found that level is reduced compared to the control group (Figure S14). These results suggest that intracellular calcium level depends on regulation of Nfil3 protein.

## Discussion

In the present study, we showed that mouse Nfil3 mRNA has an IRES element in the 5′-UTR and is translated to protein in a cap-independent manner. We showed that this cap-independent translation is important for the regulation of circadian rhythms^33^,^34^,^35^,^56^. We searched for a crucial ITAF and found that hnRNP A1 is involved in IRES-mediated translation of Nfil3, which is one of the mammalian clock genes. hnRNP A1 directly binds to the Nfil3 5′-UTR, and this binding is dramatically decreased in IRES element-deleted RNAs. hnRNP A1 has been studied as an ITAF in several IRES-mediated translation processes, and is considered to have dual functions, where it can act as both a positive and a negative regulator of translation^47^,^48^,^49^. In our current study, knockdown of hnRNP A1 led to a decrease in endogenous Nfil3 protein level, which could be due to a reduction in IRES-mediated translation. Moreover, hnRNP A1 downregulation in MC3T3-E1 cells dramatically diminished rhythmic Nfil3 protein oscillation, without any effect on mRNA oscillation. These results suggest that phase-dependent IRES-mediated translation of Nfil3 mRNA is a critical regulatory mechanism for robust Nfil3 protein oscillation.

We did not observe a complete reduction in IRES-mediated translation after knockdown of hnRNP A1. There are two possible reasons for this: firstly, it is attributed to the knockdown efficiency of hnRNP A1 shRNA. hnRNP A1 protein is abundant in the cell, and shRNA may not be possible to reduce hnRNP A1 completely, such that the remaining hnRNP A1 would be enough to drive IRES-mediated translation of Nfil3 mRNA. Secondly, among the numerous ITAFs have been identified, some of which could affect the IRES-mediated translation of Nfil3 mRNA. Further investigation into these other ITAFs would be necessary to completely understand IRES-mediated translation of Nfil3 mRNA.

According to our results, hnRNP A1 knockdown did not affect endogenous Nfil3 mRNA stability. However, hnRNP A1 is also involved in other posttranscriptional regulatory processes such as mRNA splicing and microRNA processing^45^,^46^. Because hnRNP A1 may bind to other regions of the Nfil3 mRNA, knockdown of hnRNP A1 may affect other aspects of posttranscriptional regulation of mouse Nfil3 mRNA besides IRES-mediated translation. Hence, it will therefore be necessary to consider other posttranscriptional regulatory mechanisms that may involve hnRNP A1.

The suprachiasmatic nucleus (SCN) acts as a master clock that communicates systemic time-of-day information to peripheral tissues, including the musculoskeletal system^4^,^5^. A robust circadian rhythm of endogenous Nfil3 protein and mRNA expression have been reported in the SCN and liver^20^, and Nfil3 mRNA has a rhythmic profile in mouse osteoblasts^37^. A number of recent studies have focused on circadian rhythm in bone tissue. Mice with mutations in core clock genes such as Period 2, showed changes in bone mass, osteoblast number and osteoclast activity^57^,^58^. Nfil3 is a negative regulator of Period 2 and other D-box element-containing clock genes. Nfil3 is also reported to regulate Bmp4 transcription^37^, an important bone metabolism regulator. Bmp4 is a member of TGF-β superfamily that activates the serine/threonine kinase receptor Bmpr1a^59^ and regulates several physiological processes in bone. This suggests that Nfil3 could regulate the transcription of D-box element-containing clock and clock-controlled genes and thus elicit bone metabolism.

Endogenous calcium phosphate is directly linked to bone mineralization^53^,^54^. In our current study, we observed that downregulation of endogenous Nfil3 protein led to a decrease in intracellular calcium level. This reduction could be due to an increase in prostaglandin-endoperoxide synthase 2 (Ptgs2) and prostaglandin E2 (PGE2). Ptgs2 is a rate-limiting enzyme for PGE2, which regulates mesenchymal stem cell differentiation into the osteoblast lineage^60^ and osteoclast formation^61^. Ptgs2 and PGE2 levels are upregulated, and intracellular calcium levels are subsequently decreased, when calcium efflux is induced in bone under acidic conditions^62^,^63^. Because Nfil3 is known to negatively regulate Ptgs2 transcription^64^,^65^, our findings may provide a new link between the Nfil3 translation and Ptgs2 metabolism in bone tissue.

During IRES-mediated translation in the SCN and peripheral tissues, malfunction of hnRNP A1 could lead to diminished Nfil3 protein oscillation, which could then affect other clock genes *in vivo*. Nfil3 is also involved in neuroregeneration, immune modulation and many other physiological responses. Thus, posttranscriptional regulation, and in particular, the IRES-mediated translation of Nfil3 discovered in the present study, could provide new insights into the effect of Nfil3 in many physiological conditions, including bone metabolism.

IRES-mediated translation was first discovered in viruses, but since then, many cellular IRES-mediated translations have been identified and studied. The present study has identified a novel role for hnRNP A1 in the IRES-mediated translation of Nfil3 in the rhythmic oscillation. The circadian transcription-translation feedback loop consists of clock genes, and Nfil3 is involved in this loop, further fine-tuning mechanism of Nfil3 protein synthesis by hnRNP A1 could contribute to the further circadian clock related and many other diverse physiological phenomena.

## Materials and Methods

### Plasmid constructs and Protein purification

To generate pRF-Nfil3, mouse Nfil3 (accession no. O08750) 5′-UTR was amplified using *Pfu* polymerase (SolGent) with specific primers and the sequence was confirmed by sequencing. To generate the reverse construct pRF-Nfil3-Rev, mouse Nfil3 5′-UTR was amplified using the specific primers. To generate serial deletion constructs, Nfil3 5′-UTR fragments were amplified using the specific primers for each deletion. All PCR products were digested with SalI/SmaI restriction enzymes (BEAMS) and inserted into the intercistronic region of a pRF bicistronic vector^56^. For the promoter-deleted pRF construct, the CMV promoter was removed from pRF-Nfil3 by digestion with BglII and NheI, then self-ligated. To create a hairpin-harboring construct, a palindromic sequence was inserted at the NheI site upstream of pRF-Nfil3. For the *in vitro* binding analysis, UV-cross linking and biotinylation assay, full-length and deletion fragments of the Nfil3 5′-UTR were amplified as previously described. PCR products were digested with EcoRI/XbaI and subcloned into pBluescript SK(+) (pSK) to generate pSK-Nfil3, pSK-129, pSK-169 and pSK-236. To generate the bicistronic mRNA reporter for mRNA transfection, pCY2-RF-Nfil3, pCY2-129, pCY2-RF-169 and pCY2-RF-236 were constructed as follows: the pRF-Nfil3 and deletion constructs were digested with SalI/BamHI and inserted into pCY2-RF. To generate a FLAG-tagged hnRNP A1 overexpression vector, the coding sequence of hnRNP A1 was amplified with specific primers, then digested with SalI/BamHI and inserted into a pFALG-CMV2. All of sequences of forward and reverse primers for PCR are shown in Table S1. The pCMV·SPORT-β-gal (Invitrogen) vector was co-transfected for the normalization of reporter transfection efficiency. hnRNP A1 protein was obtained using the IMPACT^TM^-CN system (New England Biolabs). Purification of hnRNP A1 protein was performed as previously described^66^.

### Cell culture and drug treatment

MC3T3-E1 cells were cultured in Minimum Essential Medium Eagle, Alpha Modification (α-MEM) (Gibco) containing 10% fetal bovine serum (Hyclone) and 1% penicillin/streptomycin (WELGENE) at 37 °C under 95% air–5% CO_2_ atmosphere. The α_1_-adrenergic receptor (AR) pathways were stimulated using the nonspecific α_1_-AR agonist phenylephrine (Sigma-Aldrich). When the cells reached confluence, the medium was exchanged with medium containing 10 μM phenylephrine. Cells were harvested at the indicated time points for further experiments.

To block the translation, cells were treated with 20 nM rapamycin or 100 μg/ml cycloheximide (CHX) and then harvested at the indicated time points. To block the transcription, cells were treated with 5 μg/ml actinomycin D and then harvested at the indicated time points.

### Luciferase assay and β-galactosidase assay

*Renilla* and firefly luciferase activities were determined using the Dual-Luciferase^®^ Reporter Assay System (Promega) according to the manufacturer’s instructions. IRES activity was determined from the ratio of firefly to *Renilla* luciferase activity (Thermo/Fluoroskan Ascent™ FL) according to the manufacturer’s instructions. β-galactosidase activities were determined using the chlorophenol red-β-D-galactopyranoside (CPRG) (Sigma-Aldrich) according to the manufacturer’s instructions. β-galactosidase value were determined the absorbance at 570 nm.

### RNA interference

Small hairpin RNA was designed to knockdown endogenous mouse hnRNP A1. Sequences were annealed and inserted into the pLL3.7 vector. Sense strand of hnRNP A1 shRNA is 5′-tGGACTGTATTTGTGACTAAttcaagagaTTAGTCACAAATACAGTCCttttttc-3′. For knockdown of endogenous genes, a small interfering RNA for mouse Nfil3 was designed (Bioneer) and for mouse Cacna1c was followed, as previously described^67^.

### Transient transfection

For reporter and β-galactosidase plasmids transfections, MC3T3-E1 cells were seeded onto 6-well plates 24 hours prior to transfection, at a density of 2 × 10^6^ cells per well. Transfections were performed using Metafectene (Biontex) according to the manufacturer’s instructions. For reporter mRNA transfection, MC3T3-E1 cells were seeded onto 6-well plates at a density of 2 × 10^6^ cells per well. Two micrograms of capped reporter RNA was transfected into MC3T3-E1 cells at the indicated time points using Lipofectamine^®^ 2000 (Invitrogen), according to the manufacturer’s instructions. For overexpression plasmid transfection, transfection was performed using microporator (Invitrogen/Neon^®^ Transfection System). Both plasmids (2 ug) and siRNAs (2 nmoles) were transiently introduced into 1 million cells at once using the microporator. Plasmids and siRNAs were suspended with cells in modified DPBS (Hyclone), followed by short electric pulses using gold tips (supported by manufacturer), then incubated in the suitable medium. Microporation condition was as follows; MC3T3-E1 cells, 1400 V, 30 ms, 1 pulse; 293 A cells, 1250 V, 20 ms, 2 pulses. After transfection, cells were harvested for further experiments at indicated times.

### *In vitro* RNA synthesis and *in vitro* binding assay

For the *in vitro* binding assay, [^32^P] UTP (PerkinElmer) and biotin-UTP (Promega) conjugated RNAs were transcribed *in vitro* from XbaI-linearized recombinant pSK vectors using T7 RNA polymerase (Promega). To identify proteins bound to the Nfil3 5′-UTR, biotinylated RNAs were incubated overnight with 500 μg MC3T3-E1 cytoplasmic extracts and streptavidin-beads (Pierce). Precipitated proteins were characterized using LTQ-orbitrap^68^. For UV cross-linking, 2ug of purified hnRNP A1 protein was incubated with each radio-labeled RNAs at 30 °C for 30 minutes and irradiated with UV for 15 minutes using a UV cross-linker (UVP) RNA-bound proteins were analyzed using autoradiography after separation by SDS-PAGE. For mRNA transfection, pCY2-RF vectors were linearized by digestion with EcoRI. The pCY2 plasmid contains a 20-nt poly (A) sequence upstream of the EcoRI site. Reporter mRNA was transcribed *in vitro* using SP6 RNA polymerase (Promega) in the presence of a cap analog (Promega).

### Quantitative real-time RT-PCR

Quantitative real-time RT-PCR was performed as previously described^33^. Briefly, total RNA was isolated using TRI Reagent (Molecular Research Center). RNA was reverse transcribed using the ImProm-II^TM^ Reverse Transcription System (Promega) according to the manufacturer’s instructions. For detection and quantification, a StepOnePlus Real-Time PCR System (Applied Biosystems) was used with the FastStart UniversalSYBR Green Master (Roche). Quantitative real-time PCR data were analyzed by the comparative C_T_ method. Sequences of forward and reverse primers for quantitative real-time PCR are shown in Table S2.

### Subcellular fractionation

To prepare cytoplasmic extracts, MC3T3-E1 cells were lysed at 4 °C in a hypotonic buffer containing 10 mM HEPES (pH 7.9), 1.5 mM MgCl_2_, 10 mM KCl, 1 mM DTT and 0.2 mM PMSF. After incubation on ice for 15 minutes, added 10% volume of lysis buffer containing 2.5% NP-40 in hypotonic buffer than incubate on ice for 10 minutes, cellular debris was removed by centrifugation (3,500 rpm) at 4 °C for 4 minutes. Nuclei were resuspended and spin down twice in lysis/extraction buffer to avoid contamination of cytoplasmic proteins. To prepare nuclear extracts, pelleted nuclei were placed in nuclear extraction buffer containing 20 mM HEPES (pH 7.9), 0.2 mM EDTA (pH 8.0), 1.5 mM MgCl_2_, 450 mM NaCl, 1 mM DTT, and 0.2 mM PMSF. After incubation on ice for 20 minutes, samples were centrifuged (15,000 rpm) at 4 °C for 20 minutes. The supernatants of fractionation were decanted, and protein concentration was determined using Bradford reagent (AMERSCO).

### Immunoblot analysis

MC3T3-E1 cells were lysed with TNE buffer containing 50 mM Tris (pH 7.4), 140 mM NaCl, 5 mM EDTA and a protease inhibitor tablet (Roche), followed by sonication. Protein concentration of lysates were determined using Bradford reagent (AMERSCO). Proteins were resolved by SDS-PAGE and transferred to nitrocellulose membranes (Pall corporation), incubated with blocking buffer (5% non-fat dry milk in Tris-buffered saline and 0.1% Tween 20) for 30 minutes. Immunoblots were performed using primary antibodies against NFIL3, hnRNP A1, 14-3-3ζ, LAMIN B (Santa Cruz), Phospho-RPS6 (Ser 235/236) (Cell Signaling), ACTIN (MPBIO), GAPDH (Millipore) and FLAG (Sigma-Aldrich). Horseradish peroxidase (HRP)-conjugated mouse (Thermo Scientific), rabbit (Promega), rat or goat (Bethyl Laboratories) secondary antibodies were detected with SUPEX ECL reagent (Neuronex) and a LAS-4000 system (FUJI FILM), according to the manufacturer’s instructions. The integrated blot density was quantified through Image J software-based analysis (http://rsb.info.nih.gov/ij/).

### Intracellular calcium measurement

MC3T3-E1 cells were harvested at the indicated time points and lysed with a chelating agent-free lysis buffer. Intracellular calcium levels were measured using a Calcium Colorimetric Assay (Sigma-Aldrich) according to the manufacturer’s instructions.

### Statistical analysis

All quantitative data are presented as the mean ± standard error of the mean (SEM). Comparisons between two groups were analyzed by two-tailed unpaired Student’s *t*-tests. For comparisons between more than two groups, a one-way analysis of variance (ANOVA) was used with a Tukey’s test. To analyze the knockdown effects of different shRNAs on time points, a two-way ANOVA was performed, followed by Bonferroni post-test. A *P* value less than 0.05 was considered statistically significant. ns *P* ≥ 0.05, *P < 0.05, **P < 0.01, *** *P* < 0.001, ****P < 0.0001.

## Additional Information

**How to cite this article**: Kim, H.-J. *et al*. Heterogeneous nuclear ribonucleoprotein A1 regulates rhythmic synthesis of mouse Nfil3 protein via IRES-mediated translation. *Sci. Rep.* **7**, 42882; doi: 10.1038/srep42882 (2017).

**Publisher's note:** Springer Nature remains neutral with regard to jurisdictional claims in published maps and institutional affiliations.

## Supplementary Material

Supplementary Information
