# Supplementary material for: Heterogeneous nuclear ribonucleoprotein A1 regulates rhythmic synthesis of mouse Nfil3 protein via IRES-mediated translation
Source: Sci Rep. 2017 Feb 21;7:42882. doi: 10.1038/srep42882 (PMC5318856; doi:10.1038/srep42882)
Supplement: Supplementary Information [file srep42882-s1.pdf]

Supplementary information for

Heterogeneous nuclear ribonucleoprotein A1 regulates rhythmic synthesis of mouse Nfil3 protein via IRES-mediated translation

Hyo-Jin Kim[1], Hwa-Rim Lee[2], Ji-Young Seo[3], Hye Guk Ryu[1], Kyung-Ha Lee[4], Do-Yeon Kim[5] and Kyong-Tai Kim[1, 3, *]

[1]Department of Life Sciences, Pohang University of Science and Technology (POSTECH), Pohang, Gyeongbuk, Korea; [2]School of Interdisciplinary Bioscience and Bioengineering, Pohang University of Science and Technology (POSTECH), Pohang, Gyeongbuk, Korea; [3]Division of Integrative Biosciences and Biotechnology, Pohang University of Science and Technology (POSTECH), Pohang, Gyeongbuk, Korea; [4]Division of Bio-Technology and Convergence, Daegu Haany University, Gyeongsan, Gyeongsangbuk-do, Korea; [5]Department of Pharmacology, School of Dentistry, Kyungpook National University, Daegu, Korea

* To whom correspondence should be addressed.

Tel: 82-54-279-2297

Fax: 82-54-279-2199

Email: ktk@postech.ac.kr

Supplementary Figures

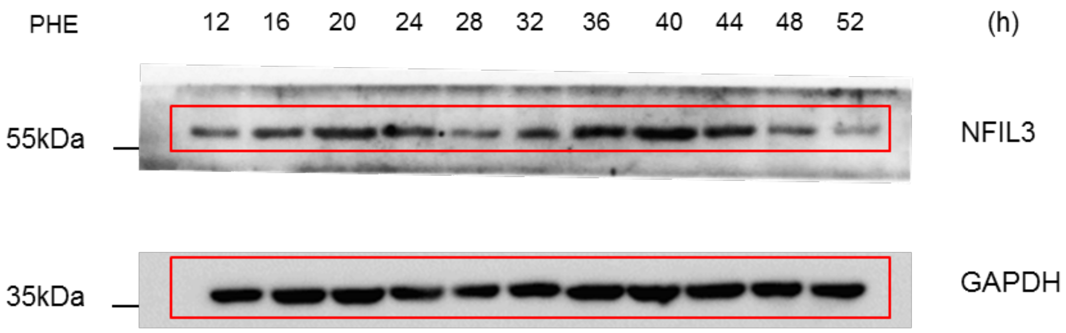

Figure S1. The full-length gel blots that included in Figure 1a.

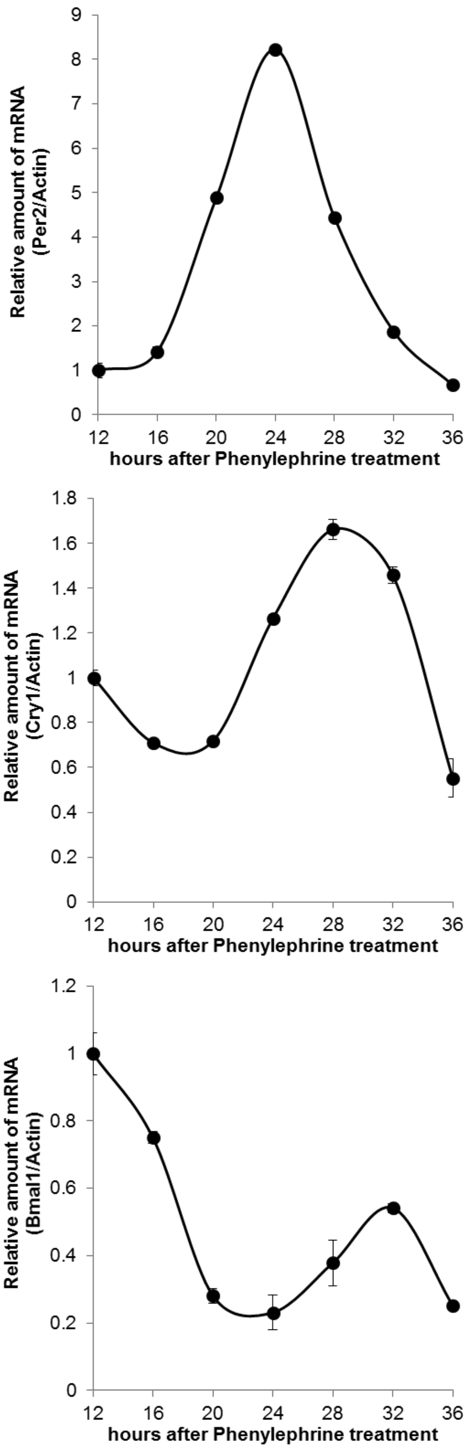

Figure S2. Core clock gene oscillation after PHE treatment. MC3T3-E1 cells were treated with 10 μM phenylephrine (PHE). After 12 hours, cells were harvested at the indicated time points and subjected to quantitative real-time PCR analysis. Relative Bmal1, Cry1 and Per2 mRNA levels were normalized to Actin mRNA. RNA levels at the initial time point were arbitrarily set to 1.0.

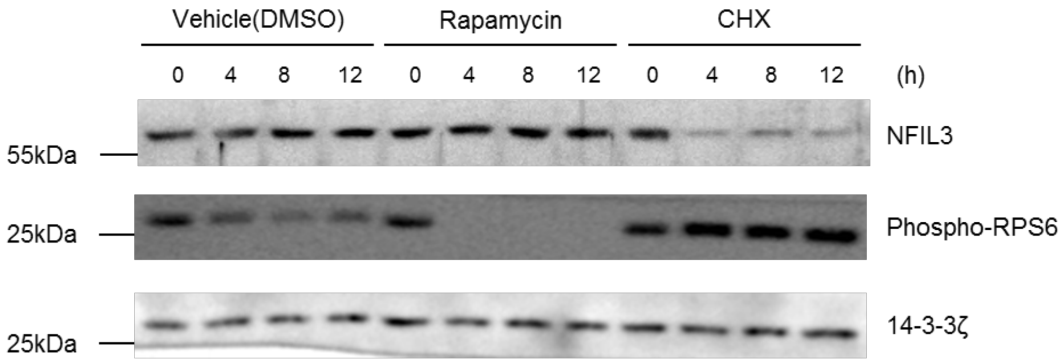

Figure S3. Nfil3 protein synthesis is rapamycin-insensitive. Rapamycin and cyclohexamide (CHX) treated MC3T3-E1 cells were harvested at the indicated time points. Protein levels were measured by immunoblotting.

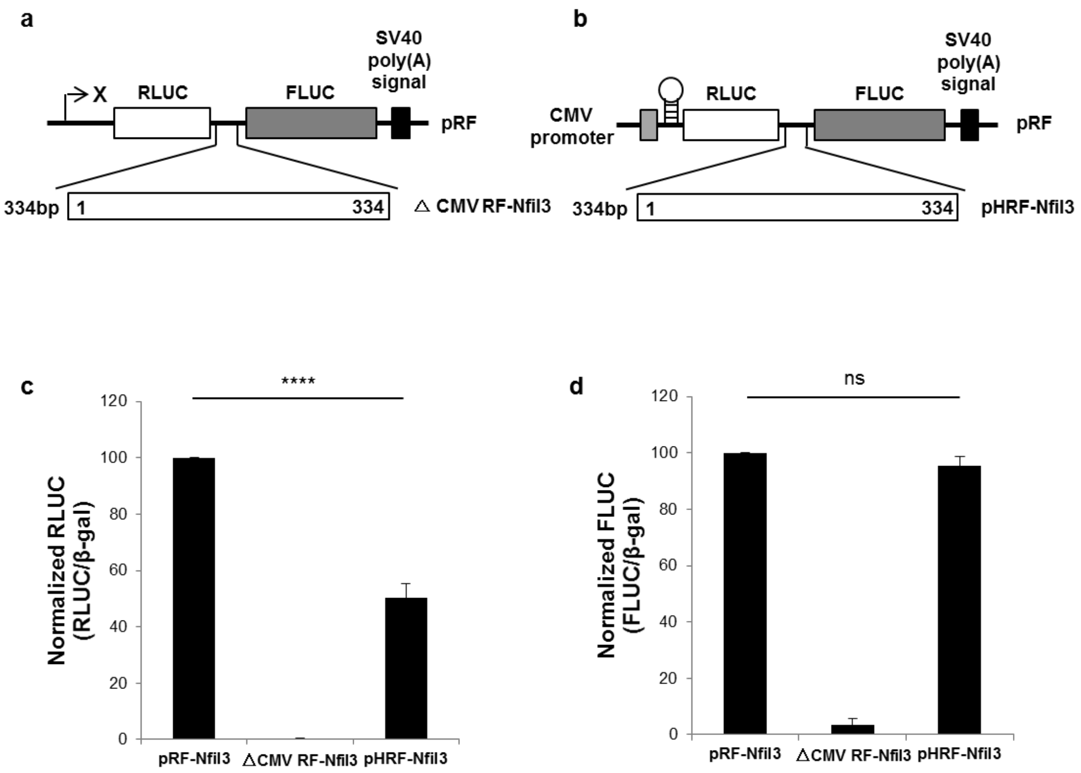

Figure S4. Normalized luciferase activities of Nfil3 5'-UTR. (**a**) Schematic diagram of the bicistronic reporter vector with no CMV promoter. (**b**) Schematic diagram of the bicistronic reporter vector harboring a hairpin structure. (**c,d**) Reporter vectors without the CMV promoter or hairpin structure were transfected into MC3T3-E1 cells. Activities of Nfil3 5'-UTR containing the reporter vectors were calculated based on the ratio of each luciferase, and normalized luciferase activities of pRF-Nfil3 were set to 100 (n = 3), **** $P < 0.0001$. The plasmid containing β-galactosidase was co-transfected for the normalization.

a

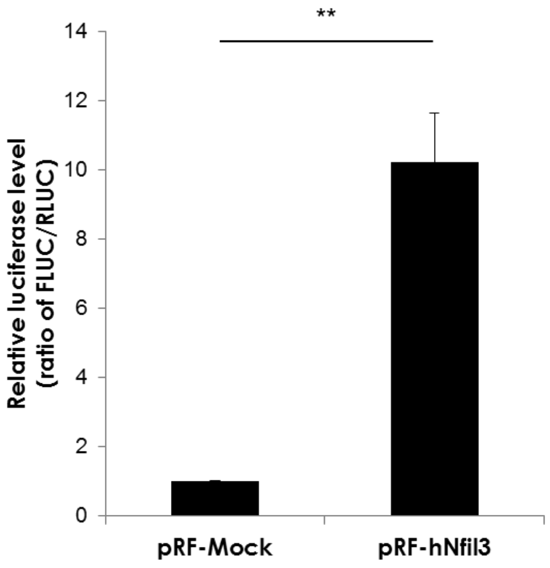

b

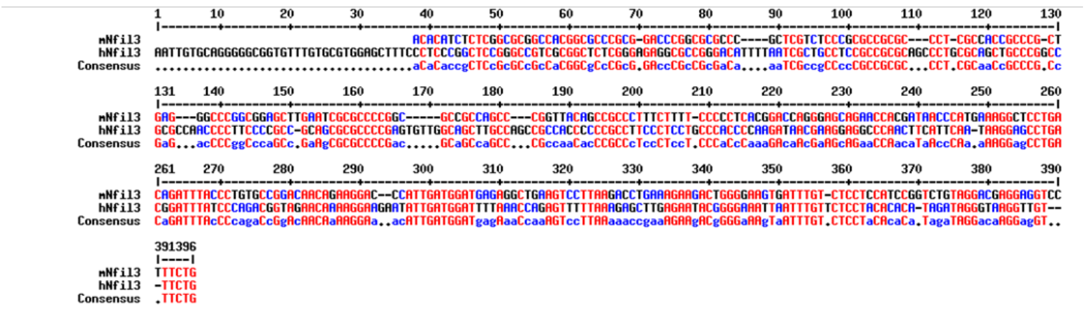

Figure S5. Human Nfil3 5’-UTR also have IRES activity. (**a**) 293A cells were transfected with a bicistronic reporter vector. Samples were harvested 24 hours after transfection of the reporter vector then subjected to a luciferase assay. The ratio of FLUC/RLUC of cells transfected with pRF empty vector was set to 1.0. Error bars represent mean ± SEM (n = 3), ** $P < 0.01$. (**b**) Sequence alignment of human (NCBI accession number: NM_001290000) and mouse Nfil3 5’-UTRs.

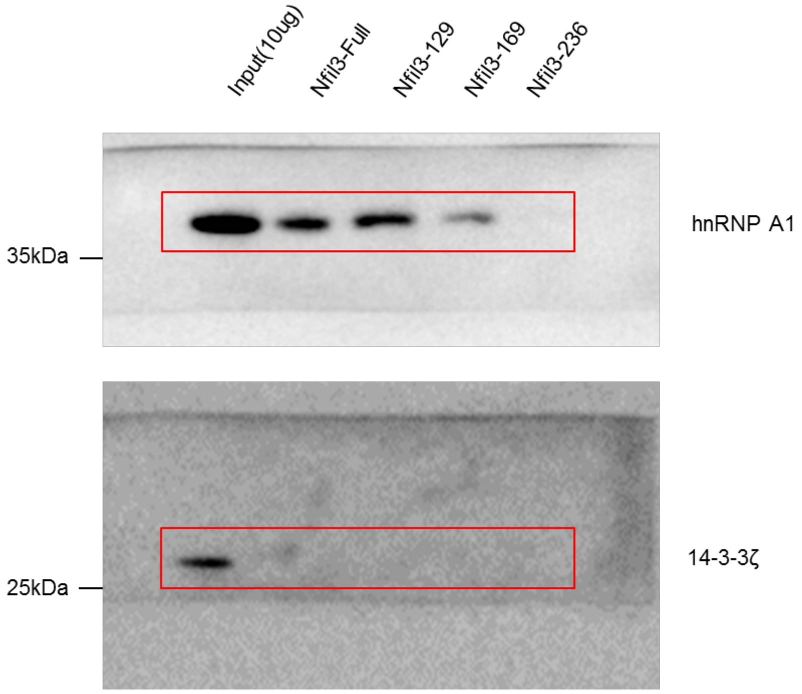

Figure S6. The full-length gel blots that included in Figure 4b.

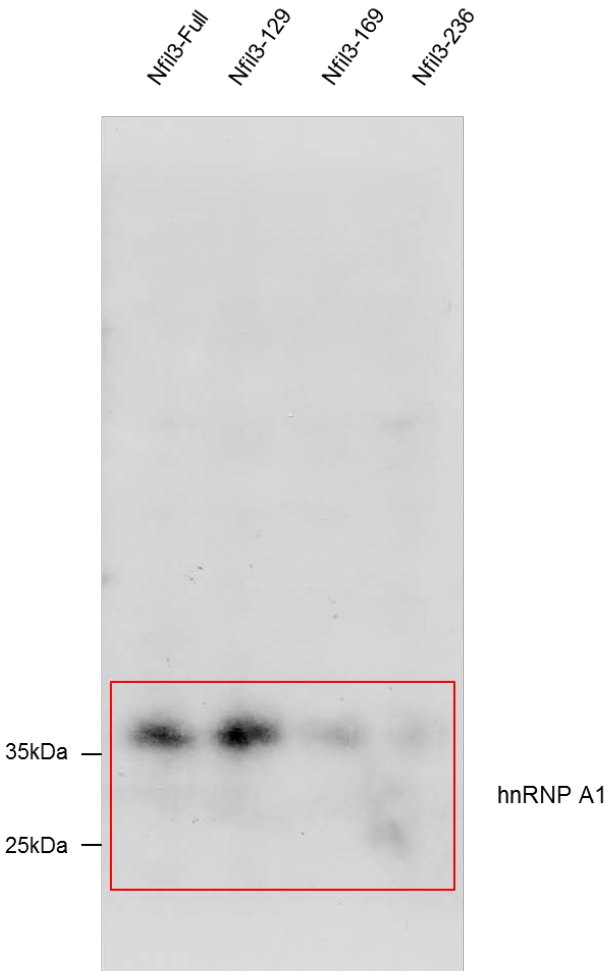

Figure S7. The full-length gel blot that included in Figure 4c.

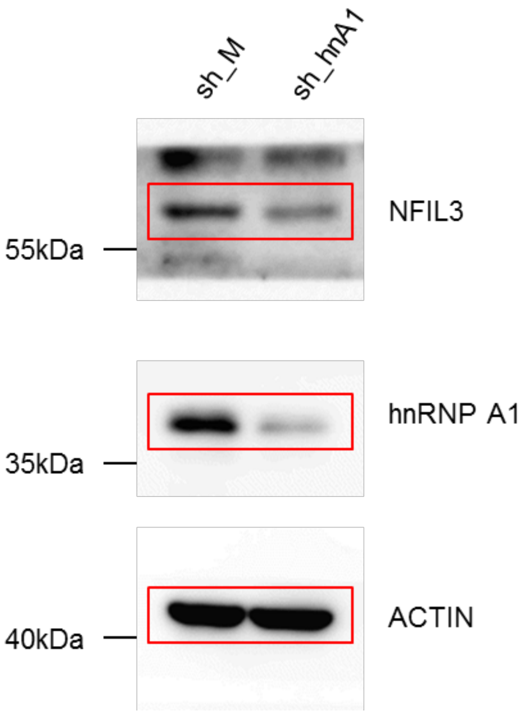

Figure S8. The full-length gel blots that included in Figure 5a.

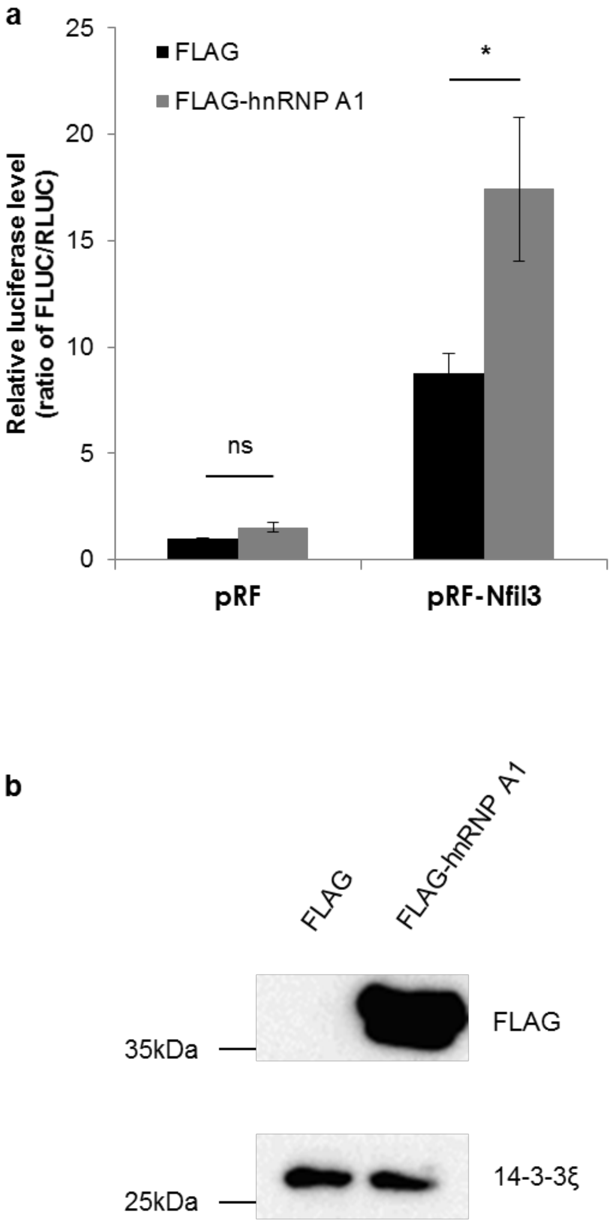

Figure S9. Overexpression of hnRNP A1 upregulates Nfil3 IRES activity. (**a**) 293A cells were transfected with FLAG and FLAG-hnRNP A1 before transfection with a bicistronic reporter vector. 293A cells were used to ensure efficient overexpression. Samples were harvested 24 hours after transfection of the reporter vector then subjected to immunoblotting and a luciferase assay. The ratio of FLUC/RLUC of cells transfected with FLAG and pRF empty vector was set to 1.0. Error bars represent mean ± SEM (n = 4), * $P < 0.05$. (**b**) Confirmation of hnRNP A1 overexpression by immunoblotting.

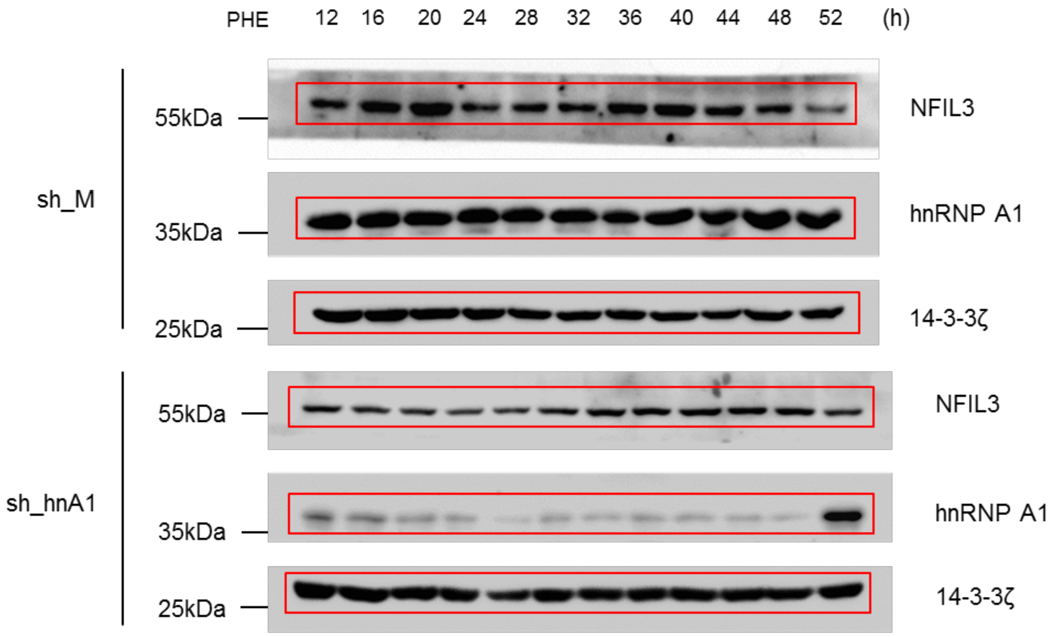

Figure S10. The full-length gel blots that included in Figure 6a.

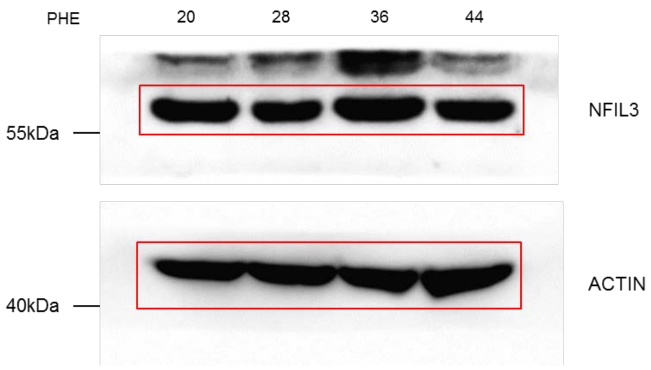

Figure S11. The full-length gels blots that included in Figure 7a.

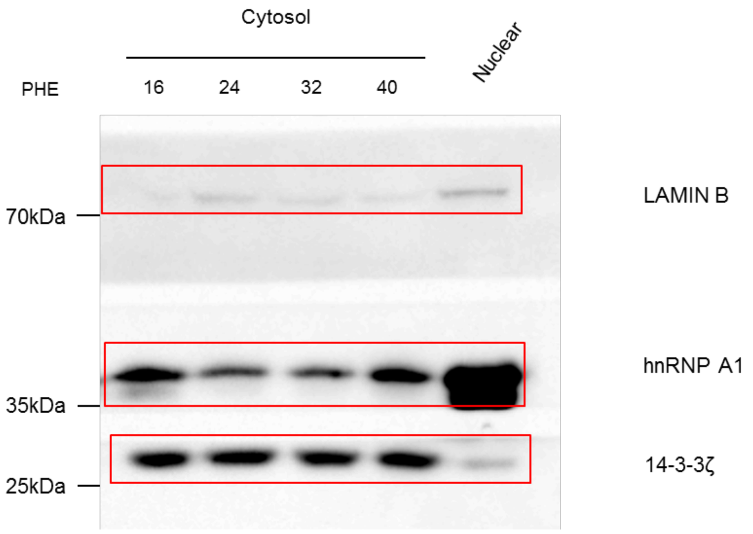

Figure S12. The full-length gels blots that included in Figure 7b.

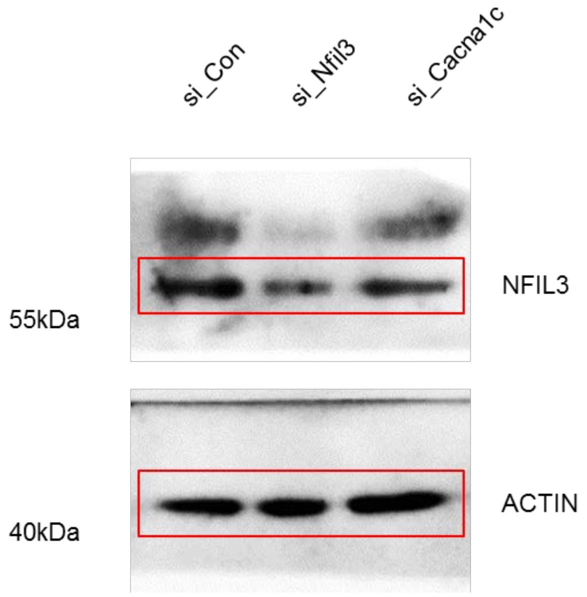

Figure S13. The full-length gels blots that included in Figure 8a.

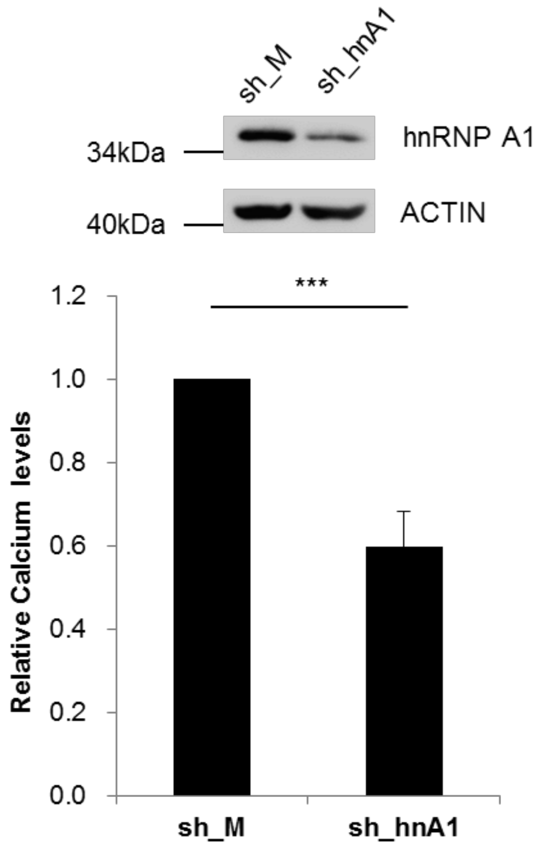

Figure S14. Knockdown of hnRNP A1 affects to intracellular calcium level. sh_M and sh_hnA1 expression vectors were transfected into MC3T3-E1 cells. Cells were harvested 48 hours after transfection and subjected to immunoblotting and intracelluler calcium measurement. The intracellular calcium level in sh_M transfected cells was set to 1.0. Error bars represent mean ± SEM (n = 4), *** P < 0.001.

Supplementary Tables

	**Sequence (5' to 3')**
**pRF-Nfil3 (Forward)**	AAGTCGACACACATCTCTCGGCGC
**pRF-Nfil3 (Reverse)**	AACCCGGGCAGAAAGGACCTCC
**pRF-Nfil3-Rev (Forward)**	AACCCGGGACACATCTCTCGGCGC
**pRF-Nfil3-Rev (Reverse)**	AAGTCGACCAGAAAGGACCTCC
**pRF-129 (Forward)**	AAGTCGACGTTACAGCCGCCCTTTC
**pRF-129 (Reverse)**	Same to pRF-Nfil3 (Reverse)
**pRF-169 (Forward)**	AAGTCGACGCAGAACCACGATAA
**pRF-169 (Reverse)**	Same to pRF-Nfil3 (Reverse)
**pRF-236 (Forward)**	AAGTCGACATTGATGGATGAGAG
**pRF-236 (Reverse)**	Same to pRF-Nfil3 (Reverse)
**pSK-Nfil3 (Forward)**	AAGAATTCACACATCTCTCGGCGC
**pSK-Nfil3 (Reverse)**	AATCTAGACAGAAAGGACCTCC
**pSK-129 (Forward)**	AAGAATTCGTTACAGCCGCCCTTTC
**pSK-129 (Reverse)**	Same to pSK-Nfil3 (Reverse)
**pSK-169 (Forward)**	AAGAATTCGCAGAACCACGATAA
**pSK-169 (Reverse)**	Same to pSK-Nfil3 (Reverse)
**pSK-236 (Forward)**	AAGAATTCATTGATGGATGAGAG
**pSK-236 (Reverse)**	Same to pSK-Nfil3 (Reverse)
**pFLAG-hnRNP A1 (Forward)**	AAGTCGACATGTCTAAGTCCGAGT
**pFLAG-hnRNP A1 (Reverse)**	AAGGATCCTTAGAACCTCCTGCCA

Table S1. Sequences of PCR primers.

	Sequence (5' to 3')
**mNfil3 (Forward)**	ATGAGGGTGTAGTGGGCAAG
**mNfil3 (Reverse)**	GTTCACTTCCGGAACCTTCA
**mPeriod2 (Forward)**	AGGATGTGGCAGGTAACAGG
**mPeriod2 (Reverse)**	ATGCTCCAAACCACGTAAGG
**mCryptochrome1 (Forward)**	GCCAGGCGGAGAAACTGAA
**mCryptochrome1 (Reverse)**	AAAATTTGCCACCCAAGCTTT
**mBmal1 (Forward)**	GCAGTGCCACTGACTACCAAGA
**mBmal1 (Reverse)**	TCCTGGACATTGCATTGCAT
**mCacna1c (Forward)**	TTGCCCTTCTTGTGCTCTTC
**mCacna1c (Reverse)**	TATGCCCTCCTGGTTGTAGC
**mGapdh (Forward)**	GCCATCAACGACCCCTTCATT
**mGapdh (Reverse)**	GCTCCTGGAAGATGGTGATGG
**mβ-actin (Forward)**	GGCACCACACCTTCTACAATG
**mβ-actin (Reverse)**	GGGGTGTTGAAGGTCTCAAAC

Table S2. Sequences of quantitative real-time PCR primers.
